# “En-Face” Spectral-Domain Optical Coherence Tomography Findings in Multiple Evanescent White Dot Syndrome

**DOI:** 10.1155/2014/928028

**Published:** 2014-04-22

**Authors:** Flore De bats, Benjamin Wolff, Vivien Vasseur, Aude Affortit, Laurent Kodjikian, José-Alain Sahel, Martine Mauget-faÿsse

**Affiliations:** ^1^Department of Ophthalmology, Croix-Rousse University Hospital, Hospices Civils de Lyon, University of Medicine Lyon 1, 103 Grande rue de la Croix Rousse, 69317 Lyon Cedex 04, France; ^2^Professor Sahel Department, Rothschild Ophthalmologic Foundation, 25 rue Manin, 75019 Paris, France; ^3^Kleber Retinal Center, 50 Cours Franklin Roosevelt, 69006 Lyon, France; ^4^Monticelli Paradis Retinal Center, 88 rue du Commandant Rolland, 13008 Marseille, France

## Abstract

*Purpose*. The recent use of “en-face” enhanced-depth imaging spectral-domain optical coherence tomography (EDI SD-OCT) helps distinguish the retinal layers involved in the physiopathology of multiple evanescent white dot syndrome (MEWDS). *Methods*. Four patients presenting with MEWDS underwent a comprehensive ocular examination including C-scan (“en-face”) EDI SD-OCT at the initial visit and during follow-up. *Results*. C-scans combined with the other multimodal imaging enabled the visualization of retinal damage. Acute lesions appeared as diffuse and focal disruptions occurring in the ellipsoid and interdigitation zones. The match between autofluorescence imaging, indocyanine green angiography, and “en-face” OCT helped identify the acute microstructural damages in the outer retina further than the choroid. Follow-up using “en-face” EDI-OCT revealed progressive and complete recovery of the central outer retinal layers. *Conclusion*. “En-face” EDI SD-OCT identified the site of initial damage in MEWDS as the photoreceptors and the interdigitation layers rather than the choroid. Moreover, “en-face” OCT is helpful in the follow-up of these lesions by being able to show the recovery of the outer retinal layers.

## 1. Introduction

Multiple evanescent white dot syndrome (MEWDS) typically affects young females and presents as a sudden visual loss with paracentral scotomas. These symptoms usually occur following an influenza-like illness, but the pathophysiologic mechanism is still poorly understood. In 80% of cases, the condition remains unilateral. Fundus examination reveals small, tiny perifoveal yellow-white dots, foveal granularity, and, in some cases, very mild vitritis, vasculitis, venous dilations, and papillitis. In addition to these clinical parameters, multimodal imaging including autofluorescence imaging, fluorescein (FA) and indocyanine green angiographies (ICGA), and B-scan enhanced-depth imaging spectral-domain optical coherence tomography (EDI SD-OCT) help confirm the diagnosis. It has been suggested that the initial lesion is located in the choroid due to the numerous hypofluorescent lesions observed in the late phase of ICG. The recent use of “en-face” OCT for MEWDS, which allows a layer-by-layer view of the involved retina, adds new information in the evaluation of the associated physiopathological process [1]. In this study, the C-scan or “en-face” EDI SD-OCT scans of patients presenting with MEWDS were combined with data from classical retinal imaging, namely, fundus color photography, autofluorescence imaging, angiographies, and B-scan EDI SD-OCT in order to show the initial site of damage in MEWDS.

## 2. Method

Patients presenting with MEWDS underwent a comprehensive ocular examination with the best-corrected visual acuity (BCVA), biomicroscopy, color fundus photography, autofluorescence imaging, fluorescein (FA), and indocyanine green angiographies (ICGA). B-scan and “en-face” C-scan EDI-SD-OCT were performed with a Spectralis-HRA instrument (Spectralis HRA+OCT; Heidelberg Engineering, Heidelberg, Germany). “En-face” macular mapping was obtained with 197 transverse sections in a 5.79 × 5.79 mm^2^ central retinal area. Then tridimensional reconstruction generated by pooling these sections provided a virtual macular brick, through which 496 shifting sections in the coronal plane result in the C-scan or “en-face” OCT. Functional parameters as perimetry and microperimetry were not assessed for the patients included in this study. Informed consent was obtained as required by French bioethical legislation (CE_20130319_8_BWF), in compliance with the Declaration of Helsinki for research involving human subjects.

## 3. Results

Four patients presenting with MEWDS were included in this study. All the patients were females; the mean age at diagnosis was 27 years (range 18–48 years). All the patients were referred with a few days' history of unilateral sudden blurred vision after a previous flu-like episode. All the patients of this study presented with unilateral MEWDS. In the fellow eye, the best-corrected visual acuity, biomicroscopy, color fundus photography, autofluorescence imaging, fluorescein and indocyanine green angiographies, B-scan, and “en-face” SD-OCT were normal without subclinical changes.

**Figure 1:**
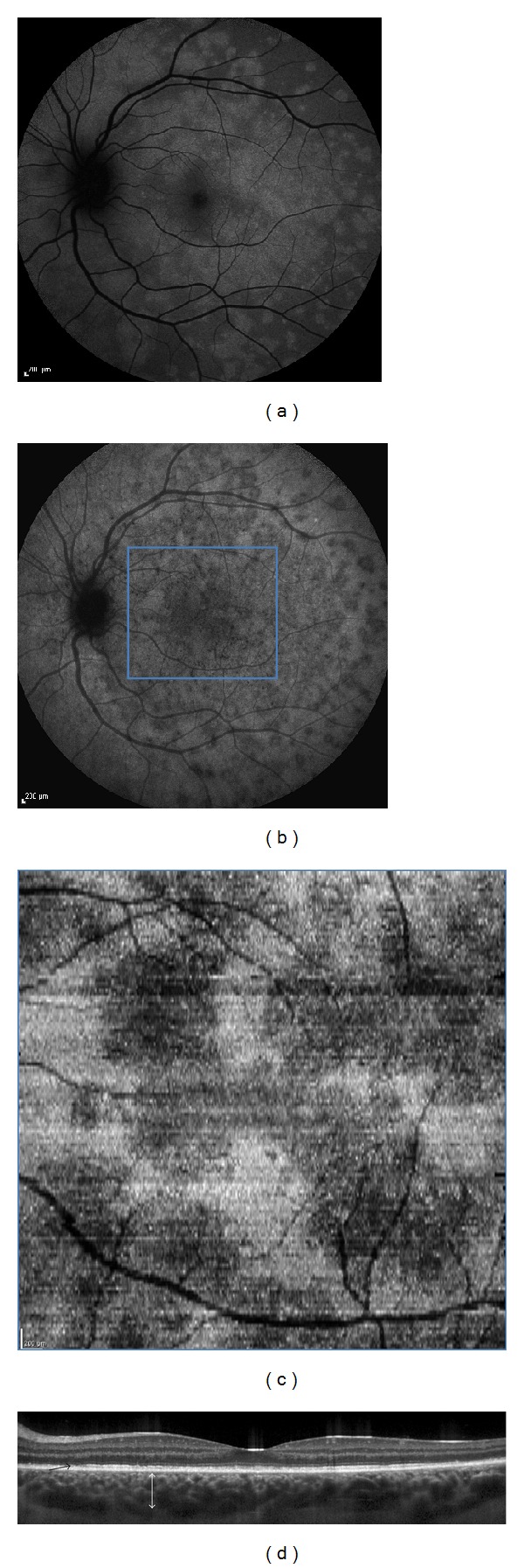
(a) Autofluorescence with hyperautofluorescent areas in the midperiphery of fundus; (b) indocyanine green angiography (ICGA) with hypofluorescent areas; (c) “en-face” EDI SD-OCT (enlarged view) at the level of the ellipsoid zone with hyporeflective areas; (d) B-scan EDI SD-OCT with disruption of the ellipsoid and interdigitation zones (black arrow). The choroid is thickened (white arrow).

In the acute phase, mean best-corrected visual acuity was 20/40 (range 20/200–20/20). Color fundus photography showed, in all patients, discrete, yellow-white dots located deep in the perifoveal and midperiphery fundus and macular granularity. In all cases, blue light autofluorescence imaging revealed multiple, more, or less confluent, hyperautofluorescent areas in the posterior pole and around the optic nerve (Figure 1(a)). FA demonstrated, in the late phase, only few slightly leaking hyperfluorescent spots. ICGA showed large, numerous, hypofluorescent areas associated with small darker points mostly located around the optic nerve (Figure 1(b)). In the midperiphery of the fundus, the areas of hypofluorescence on ICGA matched the hyperautofluorescent areas (Figures 1(a) and 1(b)).

**Figure 2:**
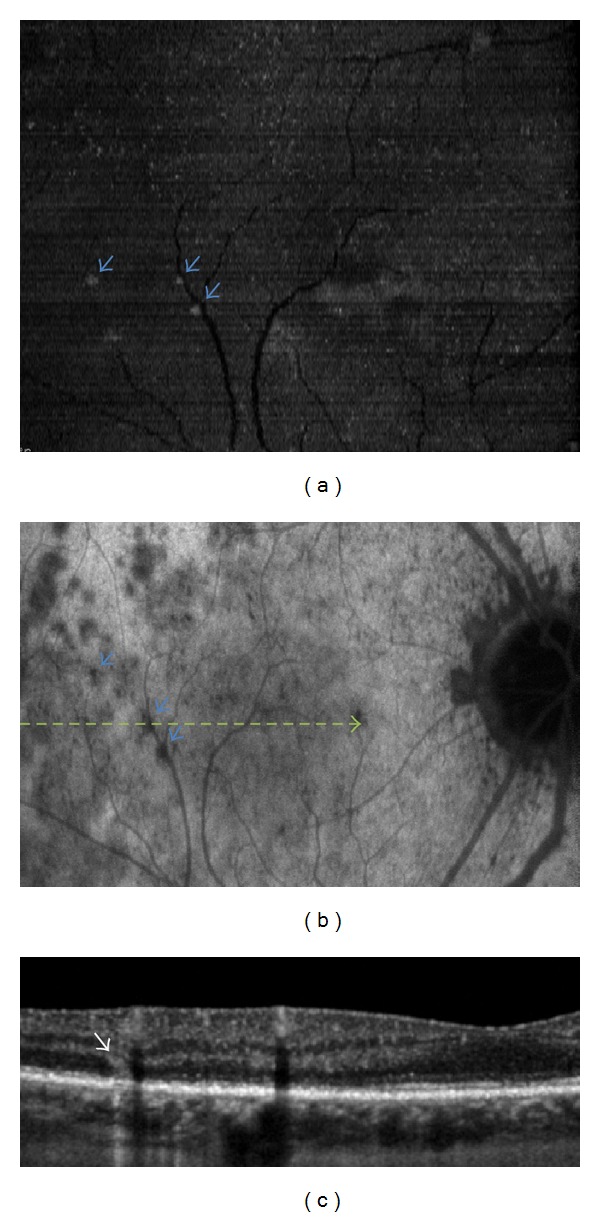
(a) “En-face” EDI SD-OCT showed hyperreflective points in the plane of the outer nuclear layer; (b) ICGA demonstrated dark points (blue arrows) matching the hyperreflective points observed on “en-face” OCT; (c) B-scan at the level of the green line showed spicule-like hyperreflective lesion (white arrows).

**Figure 3:**
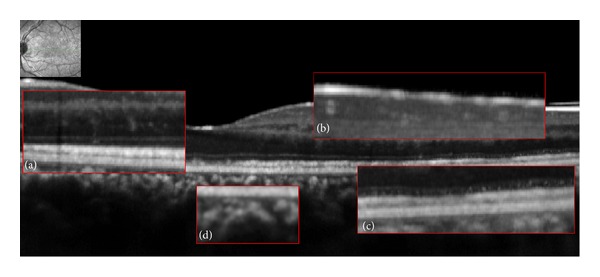
Hyperreflective points were observed in (enlarged views) (a) the outer nuclear layer, (b) the ganglion cell layer, (c) the myoid zone, and (d) the choriocapillaris.

B-EDI SD-OCT scans allowed the visualization of three distinct features of the lesions found in all four study patients. The first corresponded to diffuse and focal disruptions of the ellipsoid and interdigitation zones (Figure 1(d)). The second was represented by highly reflective, “spicule-like” lesions located between the interdigitation zone and the outer nuclear layer (Figure 2(c)). The third feature was small, round hyperreflective points located in the ganglion cell layer, the outer nuclear layer, the myoid zone, and the choriocapillaris (Figure 3).

B-EDI-OCT scans also demonstrated retrofoveolar choroidal thickening in two patients with a mean of 378 *μ*m (Figure 1(d)).

**Figure 4:**
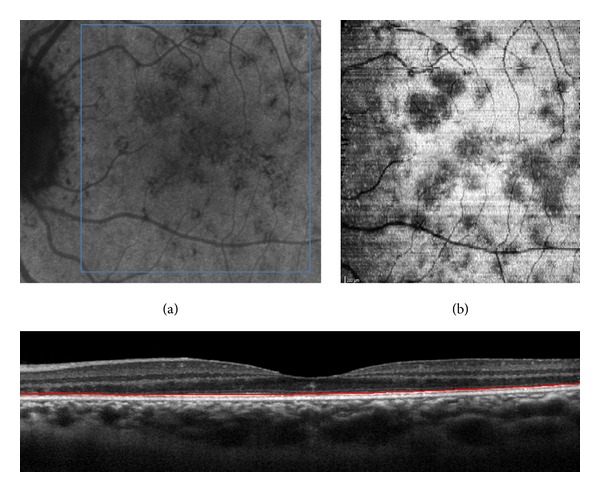
(a) Indocyanine green angiography (ICGA) with hypofluorescent areas; (b) “en-face” EDI SD-OCT (enlarged view) at the level of the ellipsoid zone with hyporeflective areas that matched the hypofluorescent areas on ICGA.

**Figure 5:**
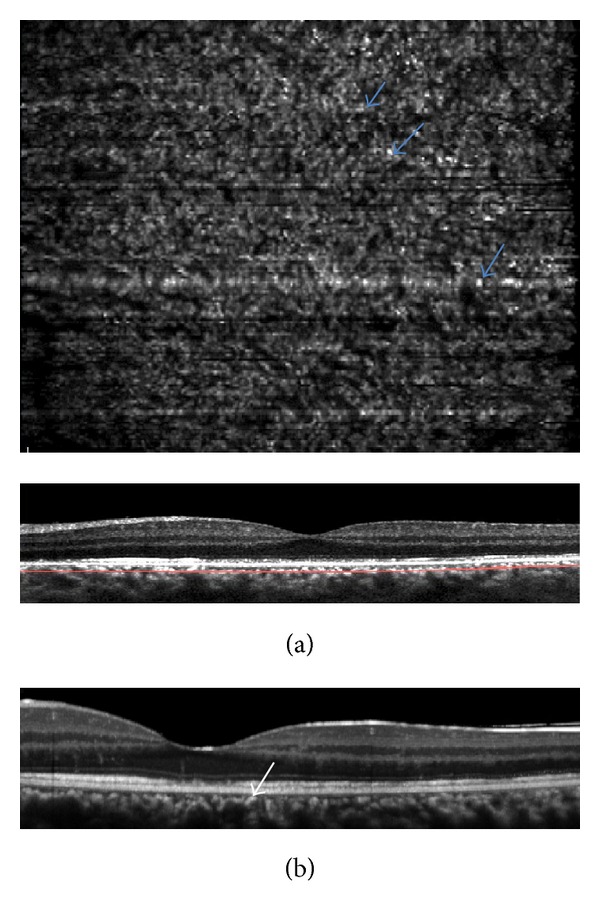
(a) “En-face” EDI SD-OCT at the level of the choriocapillaris with hyperreflective points (blue arrows) that matched (b) the hyperreflective points observed on B-scan in the choriocapillaris (white arrow).

C-EDI SD-OCT scans showed numerous round hyporeflective areas alternating with large hyperreflective areas at the level of the ellipsoid zone. The hyporeflective areas appeared confluent in the central foveal area. These hyporeflective areas corresponded to the diffuse disruption of the ellipsoid zones observed with B-scan OCT. These areas matched the hypofluorescent areas observed with ICGA in the posterior pole and in the midperiphery of the fundus (Figures 4(a) and 4(b)). The hyperreflective spicule-like lesions seen on B-scans (Figure 2(c)) were also observed with “en-face” OCT at the level of the outer nuclear layer, as hyperreflective points developed which were very obvious into this hyporeflective layer. These hyperreflective points observed on “en-face” OCT matched the small darker points seen on ICGA (Figures 2(a) and 2(b)). At the level of the choriocapillaris, “en-face” OCT showed small hyperreflective points that matched the small, round hyperreflective points observed on B-scan OCT (Figures 5(a) and 5(b)). “En-face” OCT, at the level of the choriocapillaris, did not reveal enlarged lesions as described in the plane of the ellipsoid zone and in late phase of ICGA.

**Figure 6:**
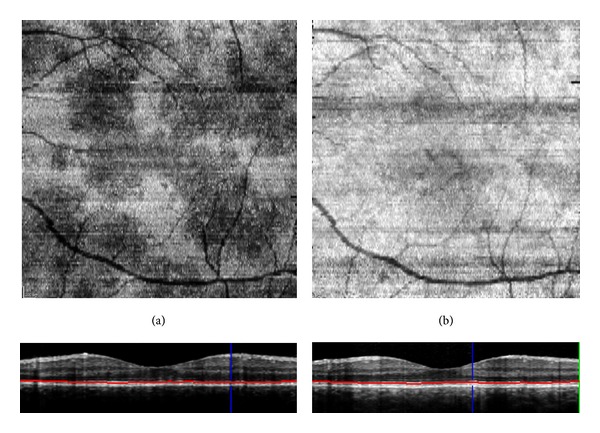
(a) “En-face” EDI SD-OCT, at the level of the ellipsoid zone in the acute phase, showing hyporeflective areas. (b) “En-face” EDI SD-OCT, at the level of the ellipsoid zone at 6-month follow-up, showing incomplete recovery of the ellipsoid zone.

Mean follow-up of the study patients was 6 months. As classically described, all the patients included in this study had a spontaneous resolution within a few weeks without treatment. Visual acuity at the final visit was 20/20 in all patients. “En-face” OCT demonstrated a progressive and incomplete recovery of the ellipsoid zone. Disappearance of the hyperreflective lesions located in the outer nuclear layer could also be observed (Figure 6). After 6 months of follow-up, the retrofoveolar choroidal thickness in B-EDI-OCT scans was nearly the same with a mean of 370 *μ*m.

## 4. Discussion

The recent use of C-scan allows, for the first time, a new and comprehensive description of acute lesions in MEWDS and a better understanding of the physiopathological process of this disease. The combined use of multimodal imaging in MEWDS involving autofluorescence imaging, ICGA, and B-scan with C-scan EDI-SD-OCT demonstrates good correspondence between the lesions observed on fundus autofluorescence and ICGA and “en-face” OCT's hyporeflective areas in the ellipsoid zone. However, the location of the initial MEWDS lesions had often been described in the choroid because of the hypofluorescent features of the lesions on ICG.

C-scan OCT, at the level of the choriocapillaris, did not reveal enlarged hypofluorescent areas corresponding to the hypofluorescent areas on ICGA. It is possible that the hypofluorescent areas on ICGA actually correspond with the hyporeflective areas in the C-scans observed in the plane of the ellipsoid zone due to earlier release of ICG molecules by the damaged RPE. The various findings from multimodal imaging suggest that the acute lesion in MEWDS is located in the outer retina and the interdigitation layers rather than in the choroid as previously described [2–4]. Thus, “en-face” OCT imaging offers a new conception of MEWDS lesions that may be located in the outer retina rather than the choroid. Similar changes in “en-face” OCT have been as well described in other types of white dots syndromes as acute posterior multifocal placoid pigment epitheliopathy (APMPPE) [1]. All these entities probably share a common pathway leading to external retinal involvement. In APMPPE, ICGA shows multiple lesions that remain hypofluorescent during all angiographic stages. As described in MEWDS, the extent of these lesions, located in the external nuclear layer, is well defined in “en-face” OCT and perfectly matches the hypofluorescent areas seen on ICGA.

Both the ellipsoid and interdigitation zones appear to be damaged in MEWDS probably because of the anatomical vicinity between the photoreceptor outer segments and the RPE apical expansions. All the cellular damages observed thanks to SD-OCT (disruption of the ellipsoid and interdigitation zones) could explain the foveal granularity feature in biomicroscopy because of a modification in the retinal transparency. After restoring the anatomical integrity of the photoreceptors, the foveal granularity often disappears.

The spicule-like lesion observed on B-scan could be due to lipofuscin leakage from the damaged RPE cells into the outer retinal layers. However, in all the study patients, hyperautofluorescence of the spicule-like lesion was not observed with autofluorescence imaging.

The small hyperreflective points, located in the ganglion cell layer, the outer retina and the choriocapillaris, and the increased choroidal thickness could represent signs of inflammation and indicate the severity of the disease. In addition, the presence of these small hyperreflective points in several locations may suggest that the inflammatory process in MEWDS possibly involves all the retinal layers and the choroid. At follow-up, retrofoveolar choroidal thickness measurement was nearly the same. Increase in choroidal thickness could be a sign of MEWDS inflammation. However, relatively great measurement variability should be considered when investigating eyes with pathologic conditions related to thick choroid.

At 6-month follow-up, in all the patients which have not been treated, “en-face” OCT demonstrated an incomplete recovery of the ellipsoid zone. It is possible that a corticosteroid treatment could have allowed larger recovering in the photoreceptors integrity.

## 5. Conclusion

“En-face” EDI SD-OCT is very helpful in the diagnosis of the acute phase of MEWDS and allows a better understanding of the associated pathophysiologic process. This technique enables a more precise description of the location and extent of structural damage occurring in this disease. “En-face” OCT associated with B-scan EDI SD-OCT suggests that the initial lesion is located in the photoreceptors and the RPE than in the choroid. Nevertheless, the associated inflammatory process appears to involve all the retinal layers and the choroid. Further prospective studies, including more patients, will be necessary to confirm these results.

## References

[1] Wolff B, Matet A, Vasseur V, Sahel J-A, Mauget-Faÿsse M (2013). B-scan and “en-face” spectral-domain optical coherence tomography imaging for the diagnosis and follow-up of white dot syndromes. *Optical Coherence Tomography*.

[2] Li D, Kishi S (2009). Restored photoreceptor outer segment damage in multiple evanescent white dot syndrome. *Ophthalmology*.

[3] Yang CS, Wang AG, Lin YH, Huang YM, Lee FL, Lee SM (2012). Optical coherence tomography in resolution of photoreceptor damage in multiple evanescent white dot syndrome. *Journal of the Chinese Medical Association*.

[4] Sikorski BL, Wojtkowski M, Kaluzny JJ, Szkulmowski M, Kowalczyk A (2008). Correlation of spectral optical coherence tomography with fluorescein and indocyanine green angiography in multiple evanescent white dot syndrome. *The British Journal of Ophthalmology*.

